# miR-155 Contributes to Normal Keratinocyte Differentiation and Is Upregulated in the Epidermis of Psoriatic Skin Lesions

**DOI:** 10.3390/ijms21239288

**Published:** 2020-12-05

**Authors:** Lucian Beer, Polina Kalinina, Martin Köcher, Maria Laggner, Markus Jeitler, Salman Abbas Zadeh, Dragan Copic, Erwin Tschachler, Michael Mildner

**Affiliations:** 1 Department of Biomedical Imaging and Image-guided Therapy, Medical University of Vienna, 1090 Vienna, Austria; lucian.beer@meduniwien.ac.at; 2 Department of Dermatology, Medical University of Vienna, 1090 Vienna, Austria; Polina.Kalinina@meduniwien.ac.at (P.K.); MartinKoecher@hotmail.de (M.K.); salman.abbaszadeh@meduniwien.ac.at (S.A.Z.); erwin.tschachler@meduniwien.ac.at (E.T.); 3 Division of Thoracic Surgery, Medical University of Vienna, 1090 Vienna, Austria; maria.laggner@meduinwien.ac.at (M.L.); dragan.copic@meduniwien.ac.at (D.C.); 4 Laboratory for Cardiac and Thoracic Diagnosis and Regeneration, 1090 Vienna, Austria; 5 Core Facility Genomics, Medical University of Vienna, 1090 Vienna, Austria; Markus.Jeitler@meduniwien.ac.at

**Keywords:** epidermal keratinocytes, keratinocyte differentiation, psoriasis, mRNA, miRNA, skin, epidermis

## Abstract

The role of microRNAs (miRNAs) during keratinocyte (KC) differentiation and in skin diseases with epidermal phenotypes has attracted strong interest over the past few years. However, combined mRNA and miRNA expression analyses to elucidate the intricate mRNA–miRNA networks of KCs at different stages of differentiation have not been performed yet. In the present study, we investigated the dynamics of miRNA and mRNA expression during KC differentiation in vitro and in normal and psoriatic epidermis. While we identified comparable numbers of up- and downregulated mRNAs (49% and 51%, respectively), miRNAs were predominantly upregulated (76% vs 24%) during KC differentiation. Further bioinformatics analyses suggested an important inhibitory role for miR-155 in KC differentiation, as it was repressed during KC differentiation in normal skin but strongly upregulated in the epidermis of psoriatic skin lesions. Mimicking the inflammatory milieu of psoriatic skin in vitro, we could show that the pro-inflammatory cytokines IL17, IL1β and INFγ synergistically upregulated miR-155 expression in KCs. Forced over-expression of miR-155 in human in vitro skin models specifically reduced the expression of loricrin (LOR) in KCs, indicating that miR-155 interferes with the establishment of a normal epidermal barrier. Together, our data indicate that downregulation of miR-155 during KC differentiation is a crucial step for epidermal barrier formation. Furthermore, its strong upregulation in psoriatic lesions suggests a contributing role of miR-155 in the altered keratinocyte differentiation observed in psoriasis. Therefore, miR-155 represents as a potential target for treating psoriatic skin lesions.

## 1. Introduction

The epidermis is a complex tissue, mainly consisting of keratinocytes (KCs) that are constantly undergoing self-renewing, differentiation and regression processes [1,2]. Epidermal stem cells, located in the bulge region of hair follicles and inter-follicular epidermis, give rise to transient amplifying KCs that migrate from the basal to the suprabasal layers, eventually forming the cornified layer [1,3]. Thereby, KCs undergo a tightly regulated differentiation program, and disturbances in this process either cause or are a consequence of multiple skin diseases with epidermal pathologies [4,5]. Molecular mechanisms orchestrating epidermal KC differentiation have been extensively investigated over the past decades [6], leading to the identification of complex regulatory networks, involving mRNAs, miRNA, long non-coding RNAs and other epigenetic mechanisms of gene regulation [7,8,9,10]. These studies have built a basis for more sophisticated in silico methods to identify novel regulators based on subtle alterations in gene expression [11].

miRNAs are endogenous, small non-coding RNAs, regulating gene expression by sequence-specific binding mainly to the 3′ untranslated region of target mRNAs [12]. The contribution of some miRNAs, including miR-203 [13], miR-23a-3p [9] and miR-30a-3p [14], to KC differentiation has previously been investigated [12,15,16]. Besides their impact on normal KC differentiation, miRNAs have also been shown to be regulated in a variety of inflammatory skin diseases. However, the contribution of miRNAs to the epidermal abnormalities observed in these diseases is still poorly understood [15,17]. 

Psoriasis is a chronic inflammatory skin disease with characteristic epidermal pathology due to perturbed KC proliferation and differentiation, ultimately leading to defects in epidermal barrier formation [18]. Several key molecules necessary for proper establishment of the epidermal barrier, such as involucrin (IVL) and loricrin (LOR), are strongly downregulated in psoriatic skin lesions [18]. The underlying regulatory mechanisms, however, are still not fully understood. Repression of gene expression by over-expressed miRNAs has already been shown to significantly contribute to the pathogenesis of psoriasis. It was demonstrated that enhanced miR-21 expression in psoriasis lesions initiates a cascade ultimately leading to increased tumor necrosis factor alpha (TNFα) release [19]. In addition, several other miRNAs have also been found to be deregulated in psoriasis, mainly targeting genes involved in cell proliferation and cell death, including miR-31, miR-125, miR-146 and miR-155 (for review, see [20]). 

miR-155 is one of the best investigated miRNAs linked to chronic skin inflammation [21,22,23,24,25]. While its function in several immune cell types, including T-helper cells, macrophages and B-cells, has been extensively studied, little is known about its role in KC differentiation and its contribution to epidermal barrier formation [26,27,28].

In the present study, we aimed to identify novel miRNA–mRNA interaction networks involved in the regulation of epidermal KC differentiation. Our analyses identified miR-155 as a key negative regulator of KC differentiation and a potential target for the treatment of psoriasis.

## 2. Results

### 2.1. Regulation of mRNA and miRNA Expression During Keratinocyte Differentiation

**Figure 1:**
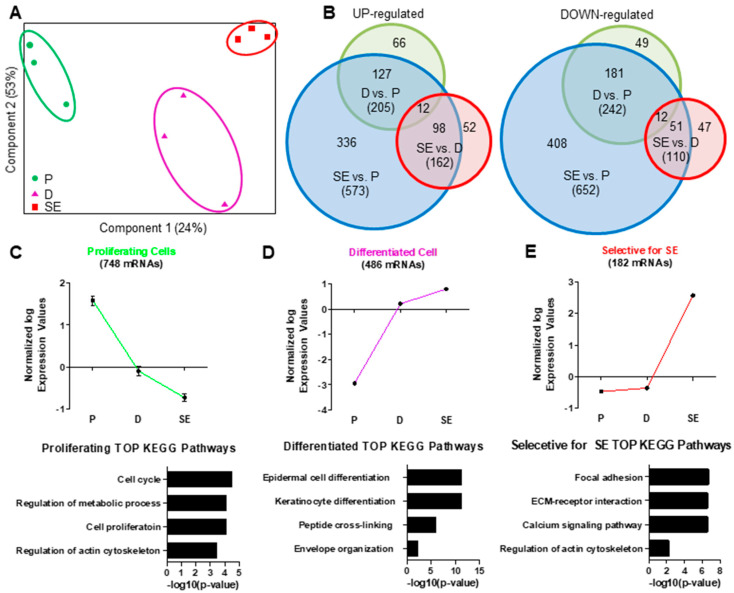
Dynamic regulation of mRNA expression during keratinocyte differentiation. (**A**) Samples are displayed based on their mRNA expression with respect to the first two components and are clustered according to their differentiation state (P proliferating monolayer; D, differentiated monolayer; SE, skin equivalent). All three conditions can be separated from each other. (**B**) Venn diagrams showing the overlap of up- and downregulated miRNAs with changes in expression between the three conditions. The area of the circles corresponds to the number of differentially expressed miRNAs. The greatest alterations were observed in mRNA expression between P keratinocytes and SE. (**C**) One cluster (green) characterizes mRNAs repressed during differentiation, (**D**) one cluster (violet) shows mRNAs upregulated during keratinocyte differentiation in monolayer and SE and (**E**) one cluster (red) shows mRNAs exclusively upregulated in SE. KEGG pathway analysis shows mRNAs of the first cluster are significantly associated with the cell cycle, while genes of the second and third clusters are associated with the epidermal differentiation and cell adhesion, respectively. ECM, extracellular matrix. *n* = 3.

In order to investigate transcriptional alterations during KC differentiation, we performed combined mRNA/miRNA expression analysis of proliferating human (pKC) and KCs differentiated in monolayer cultures (dKC) and fully differentiated skin equivalent (SE) cultures. Principal component analysis (PCA) of expressed mRNAs clearly discriminated between all three KC conditions (Figure 1A), indicating significant differences in global gene expression during KC differentiation, and also between monolayer cultures and SE. In total, 1396 mRNAs were differentially regulated between the samples, 691 (49%) of which were upregulated and 748 (51%) downregulated (Figure 1B). As expected, the highest number of differentially expressed mRNAs was observed between SE and pKC (573 upregulated and 652 downregulated mRNAs) (Figure 1B). In contrast, differentiation of KCs in monolayer cultures upregulated 205 and downregulated 242 mRNAs compared to proliferating cells. One hundred sixty-two mRNAs were up- and 110 mRNAs downregulated between dKC and SE, respectively (Figure 1B). To investigate these data sets in more detail, we first performed hierarchical clustering of the regulated mRNAs and identified three main clusters. One cluster included genes that were repressed during KC monolayer differentiation (Figure 1C). These genes were mainly associated with regulation of cell cycle, metabolic processes and actin cytoskeleton. We also found a group of genes that were upregulated during KC differentiation in both dKC and SE. These genes were primarily associated with the process of epidermal cell differentiation (Figure 1D). Genes selectively induced in SE were mainly involved in interactions with the ECM (Figure 1E), calcium signaling and remodeling of the cytoskeleton, suggesting mechanisms involved in late KC differentiation and the interaction of KCs with the ECM.

**Figure 2:**
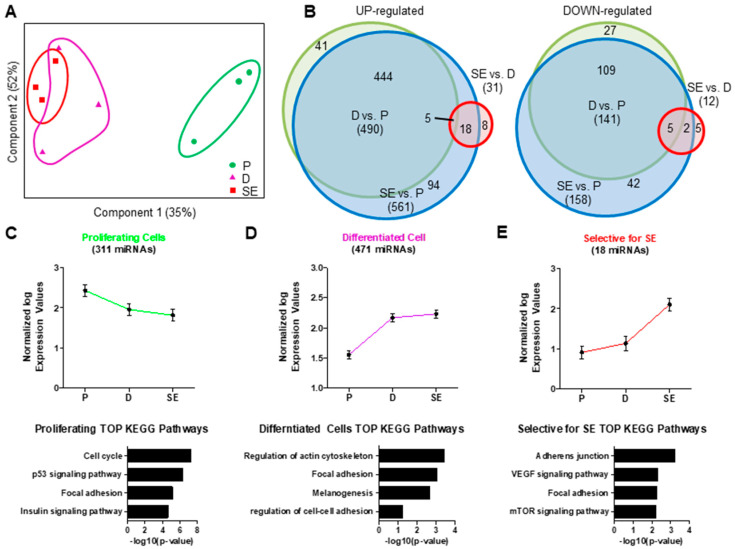
Dynamic regulation of miRNA expression during keratinocyte differentiation. (**A**) Samples are displayed with respect to the first two components and are clustered according to their differentiation state (P proliferating monolayer; D, differentiated monolayer; SE, skin equivalent). P cells can be clearly separated from D and SE indicating differences in global miRNA expression patterns. Differentiated keratinocytes cultures in monolayers and in SE cannot be clearly separated in the PCA plot. (**B**) Venn diagrams showing the overlap of up- and downregulated miRNAs with significant changes in expression between the three conditions. The area of the circles corresponds to the number of differentially expressed miRNAs. The greatest alterations were observed in miRNA expression between P keratinocytes and SE. (**C**) One cluster (green) characterizes miRNAs repressed during differentiation, (**D**) one cluster (violet) shows miRNAs upregulated during keratinocyte differentiation in monolayer and SE and (**E**) one cluster (red) shows miRNAs exclusively upregulated in SE. KEGG pathway analysis shows that miRNAs targeted mRNAs of cluster are significantly associated with cell cycle, while genes of the second and third clusters are associated with the cytoskeleton and cell adhesion, respectively. *n* = 3.

**Figure 3:**
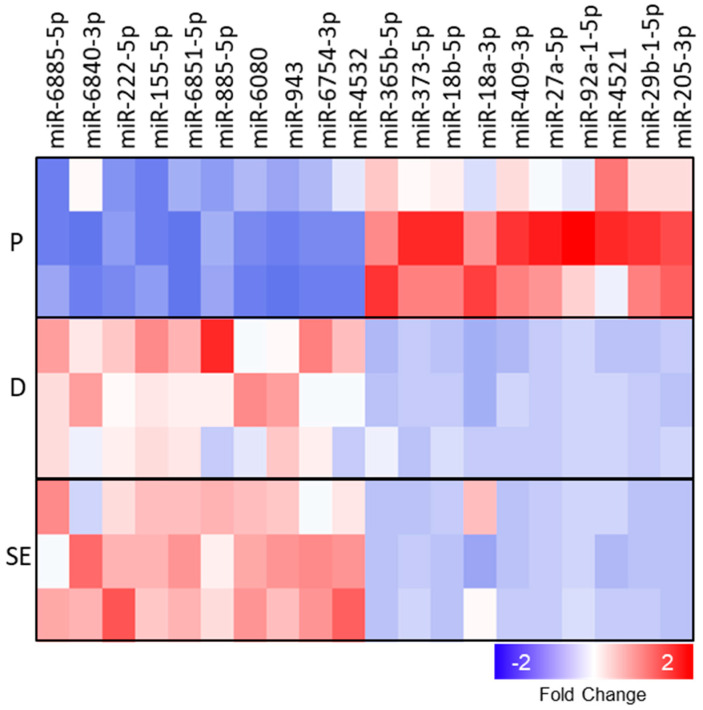
Top 10 up- and downregulated miRNAs during epidermal KC differentiation. The heatmap displays the top 10 up- and downregulated (upregulated red; downregulated blue) miRNAs differentially regulated between the three conditions. P, proliferating; D, differentiated; SE, skin equivalent. *n* =3.

Next, we analyzed miRNAs from the same samples. PCA of miRNA expression showed a clear discrimination of proliferating cells from differentiating cells and SE. In contrast to mRNA, miRNA expression profiles of dKCs and SE showed high similarity (Figure 2A). Statistical analysis revealed 800 miRNAs that were significantly regulated between the three conditions (Figure 2B), whereby 719 were differentially expressed between pKC and SE (Table S1). In general, more miRNAs were upregulated (610, 76%) than downregulated during KC differentiation (190, 24%). The ten highest differentially expressed miRNAs between pKC and SE are shown in Figure 3.

A total of 639 miRNAs were differentially expressed between pKC and dKC (496 (77%) up- and 143 (23%) downregulated, Table S2). However, only 43 miRNAs were differentially expressed between dKC and SE (31 (72%) up- and 12 (28%) downregulated, Table S3), suggesting that gene regulation by miRNA mainly occurs in the basal layers of the epidermis. Hierarchical clustering of the 800 miRNAs revealed three major clusters. One cluster included miRNAs selectively enriched in pKC (Figure 2C), one cluster contained miRNAs which were highly detectable in pKC and SE (Figure 2D), and one cluster contained miRNAs that were exclusively induced in SE (Figure 2E). To investigate possible biological functions of these miRNA clusters, we performed mRNA target prediction analysis using miRNet Gene Ontology (GO) database. miRNAs expressed in pKC and downregulated during differentiation were associated with the regulation of cell cycle, p53 signaling, focal adhesion and insulin signaling (Figure 2C). In contrast, miRNAs highly expressed in dKC and SE were associated with regulation of the cytoskeleton, focal adhesion, melanogenesis and cell–cell adhesion (Figure 2D). miRNAs exclusively expressed in SE were associated with regulation of adherens junctions, VEGF signaling, focal adhesion and mTOR signaling (Figure 2E).

### 2.2. miRNA–mRNA Interaction Networks During KC Differentiation

**Figure 4:**
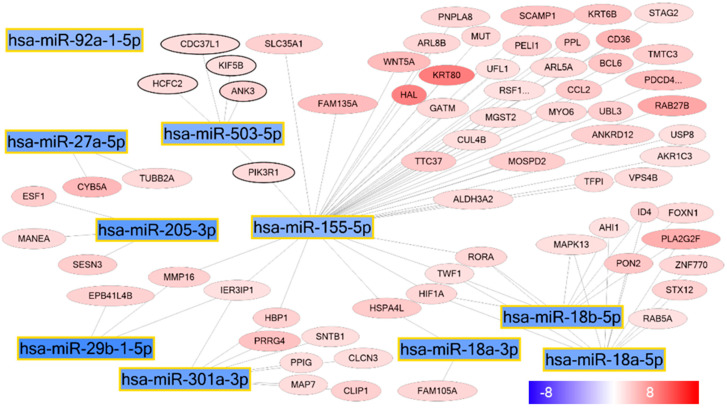
miRNA–mRNA interaction during epidermal differentiation. The interaction network displays the top 10 downregulated miRNAs during epidermal differentiation and targeted upregulated mRNAs. miR-155 encounters a central role in the network with 47 target genes.

To further investigate the role of miRNAs on epidermal KC differentiation, we selected all mRNAs upregulated and the top 10 miRNAs downregulated during KC differentiation (pKC vs. dKC) (Figure 4). Among these miRNAs, miR-155 revealed the highest number of predicted target genes, including several genes with a known function in epidermal differentiation, including *histidine ammonia-lyase* (*HAL*), *retinoic acid receptor related orphan receptor A* (*RORA*) and *keratin 80* (*KRT80*). These findings suggest that miR-155 is able to interfere with the expression of differentiation-associated genes in the basal layers of the epidermis, thereby affecting KC differentiation. 

### 2.3. Over-Expression of miR-155 in a Human Skin Model Affects KC Differentiation

**Figure 5:**
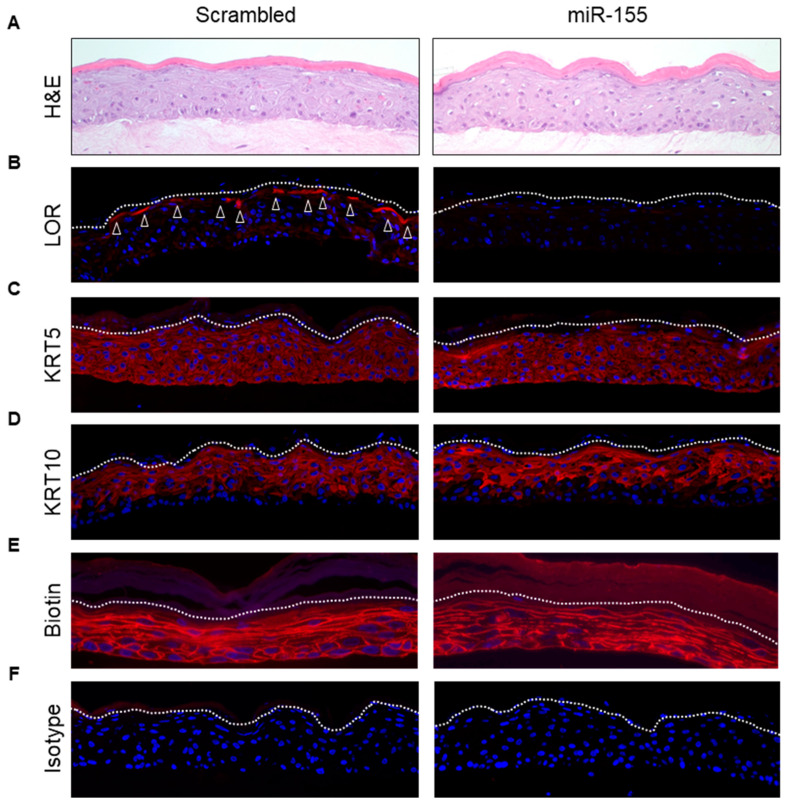
miR-155 overexpression represses loricrin expression. To study the effect of miR-155 on epidermal differentiation, we performed overexpression of miR-155 in skin equivalents (SE). While miR-155 overexpressing SE showed a normal morphological structure in H&E staining (**A**), we found (**B**) that the expression of loricrin (LOR) (white arrow head) was strongly reduced compared to control samples. There were no changes in the (**C**) KRT5 and (**D**) KRT10 expression. (**E**) Biotin permeability assay shows that miR-155 over-expression did not disturb the inside-out epidermal permeability barrier. Isotype control is shown in (**F**). The dashed line represents the border between the stratum granulosum and stratum corneum. Scale bar = 40 µm. *n* = 3.

To investigate whether miR-155 indeed affects epidermal KC differentiation, we over-expressed miR-155 in KCs and generated SE by growing these cells on a collagen matrix for up to 7 days. SE overexpressing miR-155 showed normal epidermal structure without any visible morphological alterations on day 7 (Figure 5A). However, immunostaining for several differentiation markers revealed that overexpression of miR-155 significantly downregulated the expression of loricrin (Figure 5B). The expression of other differentiation markers, including KRT5 and 10, was not affected (Figure 5C,D) nor was the inside-out epidermal permeability barrier disturbed (Figure 5E). These findings demonstrate that miR-155 interferes with the last steps of normal KC differentiation by affecting loricrin expression.

### 2.4. MiR-155 Is Upregulated in the Epidermis of Psoriatic Skin Lesions

**Figure 6:**
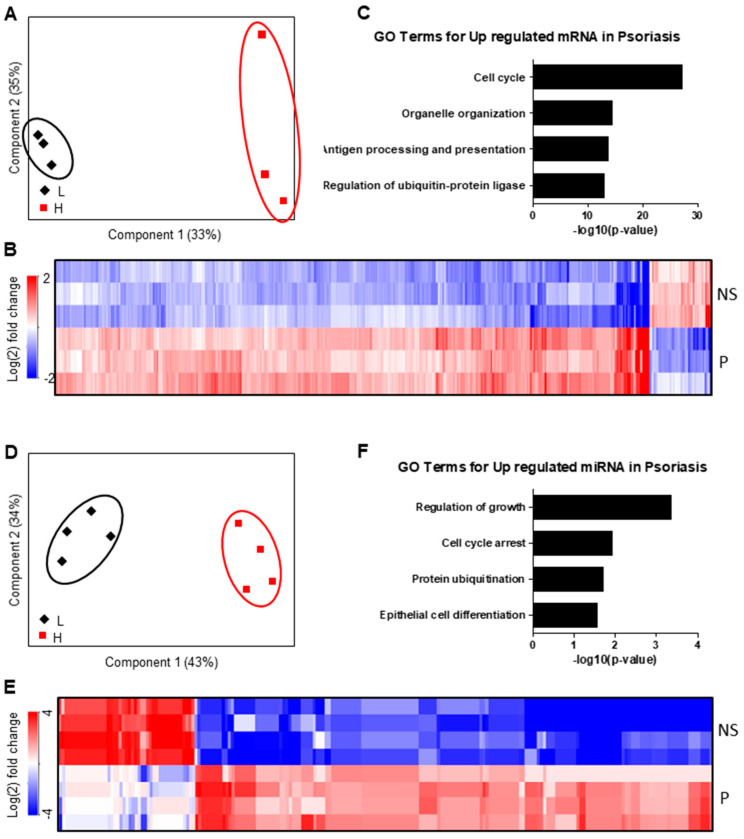
miRNA and mRNA alterations in psoriasis. (**A**) Samples are displayed based on their mRNA expression with respect to the first two components (L lesional skin; H healthy skin). The two conditions are clearly separated from each other. (**B**) The heatmap displays 141 (upregulated red; downregulated blue) significantly regulated miRNAs. (**C**) Target genes of the upregulated miRNAs in psoriasis lesions are associated with the cell cycle process and epithelial cell differentiation. (**D**) mRNA expression characteristics of the same samples are displayed in the PCA. (**E**) The heatmap shows 1987 (upregulated red; downregulated blue) differentially expressed mRNAs. (**F**) Upregulated genes in psoriasis skin are associated with the cell cycle processes. *n* = 3 for (**A**–**C**); *n* = 4 for (**D**–**F**).

To investigate changes in mRNAs and miRNAs expression in the epidermis of a skin disease with altered epidermal KC differentiation, we performed paired mRNA/miRNA expression analysis with RNA of the epidermis from psoriatic patients and healthy controls. PCA clearly discriminated the epidermis of psoriatic lesions from healthy controls when investigating global mRNA expression (Figure 6A) and miRNA transcriptional profiles (Figure 6D). 1987 genes were significantly regulated between the epidermis of psoriatic skin lesions and healthy skin. The majority of these genes were upregulated in psoriatic epidermis (1593, 80%), while only 394 (20%) were downregulated (Figure 6C, Table S4). As expected, functional analysis showed that the upregulated genes were mainly involved in cell cycle regulation, organelle organization, antigen processing and presentation as well as in the regulation of the ubiquitin-protein ligase (Figure 6B).

**Figure 7:**
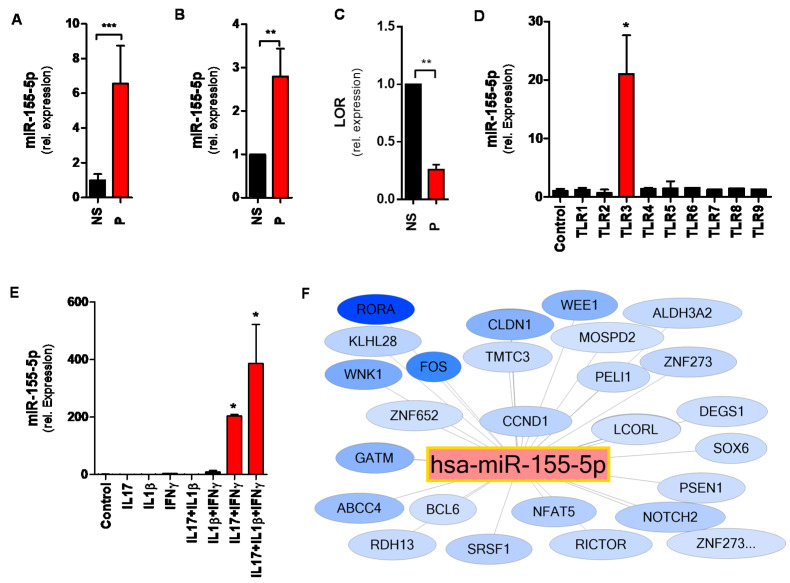
miR-155 is induced by inflammatory cytokines and TLR3 activation. (**A**) miR-155 expression is upregulated in psoriasis lesions (P) compared to normal skin (NS) based on our PCR analysis; *n* =4. (**B**) Validation data from the Genevestigator platform using expression values from five independent studies. (**C**) PCR analysis reveals significant downregulation of loricrin in psoriasis lesions (P) compared to normal skin (NS); *n* =3. (**D**) Stimulation of TLR3 induced miR-155 expression in vitro, while other TLRs did not affect miR-155 expression; *n* = 3. (**E**) The combination of IL17 and INFγ as well as IL17, INFγ and IL1β induced miR-155 expression in proliferating keratinocytes, while the individual components alone did not alter miR-155 expression; *n* = 3. (**F**) The interaction network displays miRNA-155, which is one of the top 10 upregulated miRNAs in psoriasis lesions, and their 26 downregulated, targeted mRNAs. * *p* < 0.05; ** *p* < 0.01; *** *p* < 0.001.

We also detected 141 miRNAs differentially expressed in the epidermis of psoriatic skin lesions compared to healthy controls. Eighty-seven (62%) of these miRNAs were upregulated and 54 (38%) downregulated (Figure 6F, Table S5). Functional annotations of the upregulated miRNAs showed that they were mainly involved in the regulation of cell cycle, protein ubiquitination and epidermal cell differentiation (Figure 6E). miR-155 was again one of the strongest upregulated miRNAs in the epidermis of psoriatic lesions in our transcriptome analysis (Table S5). This finding was further corroborated by quantitative real-time PCR of our samples (Figure 7A), showing a more than 6-fold upregulation of miR-155 in psoriatic epidermis compared to healthy controls. We also investigated miR-155 in a larger cohort using the bioinformatics platform Genevestigator, retrieving data from five independent studies of patients with psoriasis. Although whole skin biopsies were used in all of these studies, a significant upregulation of miR-155 was detectable in all five studies (Figure 7B). In addition, loricrin expression was significantly reduced in the epidermis of patients with psoriasis compared to normal epidermis (Figure 7C). These data indicate a potential role for miR-155 in lesional epidermis of patients with psoriasis.

### 2.5. TLR3 and Inflammatory Cytokines Upregulate miR-155 in Human Keratinocytes

Activation of pattern recognition receptors (PRRs) and the resulting immune responses in KCs have been shown to be involved in the initiation phase of psoriasis [29]. Instigated adaptive immune responses [30] induce the production and release of pro-inflammatory cytokines [31]. We therefore stimulated KCs with ligands for toll-like receptors (TLR)s 1-9 and found that only activation of TLR3 led to strong upregulation of miR-155 (Figure 7D). We next investigated whether cytokines involved in the pathogenesis of psoriasis affect miR-155 expression in primary human KCs. Stimulation of KCs with IL17, IL1β and IFNγ alone or the combination of IL1β with IL17 or IFNγ did not regulate miR-155 expression in KCs. By contrast, the combination of IL17 with IFNγ and the combination of all three cytokines strongly increased the expression of miR-155 (Figure 7E). These data suggest a contribution of TLR3 signaling and the pro-inflammatory cytokines IL17 and IFNγ to the epidermal phenotype observed in psoriasis by regulating miR-155 expression.

### 2.6. The miR-155 Network in Psoriasis

To further investigate the role of over-expressed miR-155 in the epidermis of psoriatic lesions, we generated a network with upregulated miR-155 and all mRNAs significantly downregulated in psoriatic lesions (Figure 7F). Our network revealed 26 putative target genes for miR-155 that were strongly downregulated in psoriatic skin lesions. Among them, we found genes involved in epidermal KC differentiation (*RORA*, *CLDN1*, *FOS*, *NOTCH2*), control of cell cycle (*WEE1*, *RICTOR*) and lipid metabolism (*ALDH3a2*, *DEGS1*) and transcription factors (*FOS*, *ZNF652*, *ZNF273*, *LCORL*, *SOX6*, *NFAT5*), all genes involved in cellular processes known to be compromised in psoriasis. Thus, our data suggest that targeting miR-155 represents an attractive approach for treating psoriatic skin conditions.

## 3. Discussion

Although miRNA/mRNA interaction networks in healthy and inflamed skin have been described before [19,32,33], little is known about the specific role of miRNAs in epidermal KC differentiation and their contribution to the epidermal phenotype of psoriasis. In the present study, we therefore (1) identified distinctive expression signatures and miRNA/mRNA interaction networks that dynamically map the epidermal differentiation process and (2) investigated their role in the epidermis of lesional psoriatic skin. 

Most of the available studies, addressing mRNA or miRNA expression and regulation during KC differentiation, have used KCs in monolayer culture [7,8,9,10], limiting investigations on terminal KC differentiation and epidermal barrier formation. Therefore, in our study we combined monolayer culture and organotypic skin models. This experimental setting allowed reliable bioinformatics assignment of genes related to early, late and terminal KC differentiation. In addition to terminal KC differentiation, implementation of the organotypic skin model also identified a valuable set of genes regulated by the interaction of KCs with the ECM. Strikingly, our analysis revealed that only few miRNAs were regulated between dKC and SE, suggesting a minor role of miRNAs for terminal KC differentiation and for KC–matrix interaction. The data rather suggest that epidermal miRNAs are involved in the initiation of the differentiation process, since most of the miRNAs were regulated between proliferating and early differentiated KCs. Indeed, the vast majority of miRNAs upregulated during KC differentiation target genes involved in KC proliferation, further corroborating the assumption that these miRNAs contribute to early KC differentiation by interfering with KC proliferation. This phenomenon has been reported for several miRNAs, including miR-203, which has been shown to suppress the expression of dNp63, thereby halting KC proliferation in the suprabasal layers of the epidermis [13]. In addition to many already well-investigated miRNAs [33,34,35], we identified miR-155 to be a central regulatory miRNA, expressed in proliferating KCs and repressed during KC differentiation. Our bioinformatics analysis suggests that miR-155 is involved in the regulation of several genes important for the development of a functional epidermis, such as HAL or RORA [36]. Despite this, miR-155 over-expressing skin models showed a normal epidermal morphology. This was not surprising since, although these molecules are known to fulfil important functions in the epidermis, knock-out mice or mice baring mutation of these genes exhibit a normal epidermis but develop abnormalities only in response to stress, such as ultraviolet (UV) radiation or hypoxia [37,38]. A striking finding of our study was that over-expression of miR-155 in the skin model led to a complete loss of loricrin expression. Loricin is a central protein of the cornified envelope and, therefore, important for the function of the epidermal barrier [39,40]. Although loricrin is one of proteins with the highest abundance in normal stratum corneum, loricrin-deficient mice are mostly asymptomatic [41]. This is in line with our miR-155 over-expressing skin model, where downregulation of loricrin was observed without alterations in the formation of the epidermis or barrier function. Of note, loricrin-deficient mice have also been shown to be more sensitive to UV irradiation [42], which might suggest a role of miR-155 in the stress response of the epidermis. Therefore, future studies on miR-155 over-expressing skin models will be necessary to evaluate its impact on stress, such as UV radiation. Furthermore, it remains to be elucidated whether loricrin downregulation is directly regulated by binding of miR-155 to loricrin mRNA or indirectly via the regulation of other factors. So far, bioinformatics analysis for miR-155 did not reveal a bona fide miR-155 binding site in the loricrin sequence, suggesting an indirect effect. However, to the best of our knowledge, no data are currently available describing loricin as a direct target for miRNAs, which makes literature-based bioinformatics analyses difficult.

Our study also suggests a contribution of miR-155 to the epidermal phenotype observed in psoriatic skin lesions. While few studies so far have shown an increased miR-155 expression in skin biopsies of patients with psoriasis [23,24], its contribution to the pathogenesis of this inflammatory skin disease is still unknown. Previously, infiltration of activated T-cells has been described as a major source of increased miR-155 in psoriatic lesions [43]. Our study identified, in addition to T-cells, KCs as a main source of this miRNA. Although the sole over-expression of miR-155 did not show severe morphological alterations of the epidermis in our skin model, it is conceivable that in psoriatic lesions, together with other factors (e.g., inflammatory cytokines, other miRNAs), miR-155 contributes to the epidermal pathology. We identified the pro-inflammatory cytokines IL17, INFγ and IL1β as important inducers of miR-155 in KCs, all factors well-known to contribute to the pathogenesis of psoriasis [26,27]. Our data are in line with those of Šahmatova and colleagues, who demonstrated that TNFα and INFγ alone slightly induced miR-155 expression in KCs [44]. However, we could further show that a cocktail of these cytokines, as present in psoriatic skin lesions, was much more potent in the regulation of miR-155. Our data suggest that KC-derived miR-155 is upregulated in response to these pro-inflammatory stimuli, thereby contributing to the observed epithelial phenotype [19,24,25]. In line with a previous study [45], we could also confirm a strong upregulation of miR-155 by TLR3 activation. Interestingly, all other TLR ligands tested were not able to induce miR-155 expression, suggesting a very specific response to double-stranded RNA, as present in some viruses. Although several studies reported a correlation between virus infection and psoriasis induction or progression, the underlying mechanism is still not fully understood [46]. The induction of miR-155 by viral components might therefore represent a further part of the puzzle in the pathogenesis of psoriasis.

Together, our data suggest that blocking miR-155 may be a potential strategy for the treatment of psoriasis in the future, which could have advantages over current small molecules and biologics. Targeting several factors simultaneously could potentially restore multiple pathways deregulated in the epidermis of patients with psoriasis. On the contrary, blocking miR-155 might affect other important physiological functions of this miRNA, leading to so far unpredictable problems. Therefore, more studies on miR-155 deletion in the epidermis will be necessary to fully evaluate the whole spectrum of the consequences of miR-155 inhibition.

In conclusion, we have shown that downregulation of miR-155 in the suprabasal layers of the epidermis is important for normal KC differentiation and expression of crucial elements of the skin barrier. Our finding that miR-155 is strongly upregulated within the epidermis of psoriatic lesions suggests that it contributes to the characteristic epithelial phenotype of psoriasis. In this study, we investigated one out of several interesting miRNAs identified by our analyses. Therefore, our dataset of mRNAs and miRNAs regulated during KC differentiation and in the lesional epidermis of psoriasis lays a foundation for future studies on the pathogenesis of this disease.

## 4. Materials and Methods

### 4.1. Psoriasis Samples

This study was approved by the local ethics committee (Nr. 1449/2016; 19/07/16) and conducted in accordance with the Declaration of Helsinki principles. Participants gave their written informed consent. Six millimeter punch biopsies from lesional skin of psoriatic patients were taken at the Department of Dermatology, Medical University of Vienna. Biopsies of healthy skin were obtained from abdominal skin after plastic surgery at the Division of Plastic and Reconstructive Surgery, Department of Surgery, Medical University of Vienna. The epidermis was separated from the dermis by incubation at 37 °C for 1 h with dispase II (Merck, Burlington, MA, USA). After removal, the epidermis was washed and immediately lysed in TRIzol Reagent (Invitrogen, Carlsbad, CA, USA) for RNA extraction.

### 4.2. Cell culture and Preparation of Organotypic Skin Cultures

Human primary KCs were isolated as described previously [47] and cultured in serum-free KC growth medium (KGM-2, Lonza, Basel, Switzerland). Primary human KCs from three different adult donors were used for preparation of organotypic skin cultures as described previously [48] and, in parallel, cultured in two-dimensional monolayers. For analyzing proliferating KCs, 2nd passage cells at 50% confluency were used. Differentiation was induced by maintaining confluent KCs for 7 days at high calcium (1.1 mM Ca^2+^) conditions. The quality of keratinocyte differentiation was validated by analysis of well-known differentiation markers, including keratin 1, keratin 10, the S100 genes *S100A7* and *A9*, desmoglein-1 and keratinocyte differentiation-associated protein. For in vitro assays, cytokines in the following final concentrations were used: IL-1β (10 ng/mL), TNF-α (10 ng/mL), IL-17 (10 ng/mL), INF-γ (100 ng/mL) (all R&D System, Minneapolis, MN, USA) and toll-like receptor (TLR) agonists (InvivoGen, Toulouse, France).

### 4.3. RNA Extraction and cDNA Synthesis

Total RNA from KC monolayers, skin models and epidermal sheets from healthy and psoriatic skin biopsies were extracted using TRIzol reagent according to the manufacturer’s protocol (Invitrogen). RNA concentrations were determined by a Nanodrop ND-1000 (Thermo Scientific, Waltham, MA, USA) spectrophotometer, the and quality of selected samples was assessed by Agilent 2100 bioanalyzer using RNA integrity score cut of value of > 8 (Agilent Technologies, Santa Clara, CA, USA). Samples were stored at −80 °C until further use. RNA (200 ng) of each sample was reverse-transcribed using iScript cDNA synthesis kit according to the manufacturer’s protocol (Bio-Rad, Hercules, CA, USA) in a final volume of 20 µL. cDNA was diluted with RNase/DNase-free water and stored at −20 °C until further use.

### 4.4. Quantitative Real-Time Polymerase Chain Reaction (qPCR) Analysis of miR-155

miR-155 expression was analyzed using the TaqMan^®^ MicroRNA Assay Kit (Applied Biosystems, Foster City, CA, USA). Briefly, each RT reaction contained 10 ng of total purified RNA, tem-loop RT primer, RT buffer, 0.25 mM of each dNTP, 50 U Multi-Scribe™ reverse transcriptase and 3.8 U RNase inhibitor. The reactions were incubated for 30 min at 16 °C, 30 min at 42 °C, 5 min at 85 °C and then held at 4 °C. The resulting cDNA was amplified quantitatively using LightCycler^®^ Probes Master Mix and Taqman micro-RNA assays for miR-155, and miR RNU44 as an endogenous control. The relative expression levels between samples were calculated as described below.

### 4.5. qPCR of mRNA

Relative quantification was performed as described previously [49] using the Light Cycler Master SYBR Green I kit (Roche Applied Science, Penzberg, Germany) on a Light Cycler 480 thermocycler (Roche). Primers were designed by Primer3 software (http://primer3.ut.ee/) and synthesized by Microsynth AG (Balgach, Switzerland). Samples were normalized to beta-2-microglobulin as an internal reference gene and analyzed according to the ΔΔCt method as described by Pfaffl [50]. The following primers were used: β-2-microglobulin: 5′-GATGAGTATGCCTGCCGTGTG-3′ and 5′-CAATCCAAATGCGGCATCT-3′; loricrin: 5′-GGAGTTGGAGGTGTTTTCCA-3′ and 5′-ACTGGGGTTGGGAGGTAGTT-3′.

### 4.6. miR-155 Mimics Transfection

miR-155 mimics and a scrambled control were obtained from Thermo Scientific. KCs were grown to 50–60% confluence. Fifty microliters Lipofectamine 2000 (Invitrogen) was combined with 5 mL Opti-MEM medium (Thermo Scientific) and 50 μL of the miR-155 mimics or scrambled control oligos. After incubation at room temperature for 30 min, the solution was added to 20 mL KGM-2 (Lonza) and transferred to the cells. KCs were then incubated for 24 h and used for skin models. 

### 4.7. mRNA and miRNA Microarrays

For gene expression analysis, total RNA was isolated using TRIzol reagent as described above. RNA cleanup and concentration was performed using the Rneasy MinElure Cleanup Kit (Qiagen, Venlo, Netherlands). Total RNA (200 ng) was amplified and labeled using the GeneChip^®^ WT PLUS Reagent Kit (Affymetrix, Santa Clara, CA). Fragmented and labeled samples were hybridized to either GeneChip Human Gene 2.0 ST Arrays or GeneChip Human Transcriptome 2.0 Arrays. Total RNA (200 ng) was labeled using the FlashTag Biotin HSR RNA Labeling Kit (Affymetrix) and hybridized on miRNA 4.0 Arrays (Affymetrix). Staining and scanning of the arrays were performed according to manufacturer’s protocols. Array data were submitted to the Gene Expression Omnibus database under accession number GEO: GSE145305 and GSE145059.

### 4.8. Microarray Expression Data Analysis 

The Affymetrix raw data were processed using the Transcriptome Analysis Console v.4.0 (Affymetrix). Robust multichip average (RMA) algorithm and log2-transformation were used to normalize mRNA data. A filtering step was applied in order to reduce the number of multiple hypotheses. Only transcripts were considered to be expressed if 50% of the samples in the dataset had “detected above background” >50%. An ANOVA was used to identify differentially expressed genes. *p*-values were adjusted for multiple testing using the false discovery rate (FDR) method of Benjamini–Hochberg. Probes with a FDR <5% and a >2-fold change (FC) were considered as biologically relevant. Unsupervised hierarchical clustering was applied to the microarray expression profiles, using complete linkage and Euclidian distance as the clustering methods. Paired miRNA–mRNA analysis was performed using default setting of the Transcriptome Analysis Console v.4.0 (Affymetix). Only validated miRNA–mRNA interactions were considered for our analysis.

### 4.9. Functional Annotation Clustering and Pathway Analysis

Differentially expressed mRNAs and miRNA-target mRNAs were classified according to the WEB-based Gene Set Analysis Toolkit (WebGestalt, www.webgestalt.org) as described previously [51].

### 4.10. Immunofluorescence Staining

Immunofluorescence stainings for LOR (Covance, Berkeley, CA, USA), KRT5 (Abcam, Cambridge, UK), KRT10 (Covance) and rabbit IgG isotype control (Thermofisher, Waltham, Massachusetts, USA) were performed on 5 μm sections of formalin-fixed, paraffin-embedded tissue samples as described previously [47]. 

### 4.11. Analysis of Epidermal Permeability

Punch biopsies (6 mm) of fully differentiated organotypic skin models at day 7 were placed with the dermal side downwards on a 5 μL drop of LZ-Link Sulfo-NHS-LC-Biotin (biotin, 10 mg/mL; Pierce, Rockford, IL, USA) and incubated for 60 min. After fixation, biotin was detected with streptavidin conjugated to Alexa 594 (Invitrogen-Life Technologies) on 5 µm sections. Nuclei were stained with Hoechst dye (Dako).

### 4.12. Statistical Analysis

Relative quantification of target genes was performed using the indicated reference gene according to the formula described by Pfaffl [51]. Statistical analysis was performed with GraphPad Prism V.5.01 (GraphPad Software, La Jolla, CA, USA). Comparisons between normally distributed data sets were performed by paired or unpaired t-tests. Data are displayed as arithmetic mean +/− standard deviation (SD). A two-sided *p*-value < 0.05 was considered as significant.

## Supplementary Materials

Supplementary materials can be found at https://www.mdpi.com/1422-0067/21/23/9288/s1. Table S1. miRNAs differentially expressed between pKC and SE; Table S2. miRNAs differentially expressed between pKC and dKC; Table S3. miRNAs differentially expressed between dKC and SE; Table S4. mRNAs differentially expressed between the epidermis of psoriatic skin lesions and healthy skin; Table S5. miRNAs differentially expressed between the epidermis of psoriatic skin lesions and healthy skin.

## Abbreviations

ALDH3A2	Aldehyde Dehydrogenase 3 Family Member A2
ANOVA	Analysis of variance
CLDN1	Claudin-1
DEGS1	Delta 4-Desaturase, Sphingolipid 1
dKC	Differentiated keratinocytes
ECM	Extracellular matrix
FOS	Fos Proto-Oncogene, AP-1 Transcription Factor Subunit
GO	Gene ontology
HAL	histidine ammonia-lyase
IL-1β	Interleukin-1β
IL-17	Interleukin-17
INF-γ	Interferon gamma
IVN	Involucrin
KEGG	Kyoto Encyclopedia of Genes and Genomes
KC	Keratinocyte
KRT5	Keratin 5
KRT10	Keratin 10
KRT80	Keratin 80
LCORL	Ligand Dependent Nuclear Receptor Corepressor Like
LOR	Loricrin
miRNA	microRNA
mTOR	mammalian target of Rapamycin
NFAT	Nuclear factor of activated T-cells
NOTH2	Neurogenic Locus Notch Homolog Protein 2
NS	Normal skin
PCA	Principal component analysis
pKC	Proliferating keratinocyte
PRR	Pattern recognition receptor
RICTOR	RPTOR Independent Companion Of MTOR Complex 2
RORA	Retinoic acid receptor related orphan receptor A
SE	Skin equivalent
SOX6	SRY-Box Transcription Factor 6
TLR	Toll-like receptor
TNFα	Tumor necrosis factor alpha
VEGF	Vascular endothelial growth factor
WEE1	WEE1 G2 Checkpoint Kinase
ZNF273	Zinc Finger Protein 273
ZNF652	Zinc Finger Protein 652
